# Simultaneous Percutaneous Coronary Intervention (PCI) and Endovascular Aneurysm Repair (EVAR): A Preliminary Report

**DOI:** 10.3390/jcm13185545

**Published:** 2024-09-19

**Authors:** Priscilla Nardi, Valerio Rinaldi, Maria Ludovica Costanzo, Rocco Pasqua, Francesco Loiacono, Piergaspare Palumbo, Fabio Miraldi, Gaetano Tanzilli, Vito D’Andrea, Giulio Illuminati

**Affiliations:** 1 Department of Surgery, Sapienza University of Rome, Viale Regina Elena, 324, 00161 Rome, Italy; valerio.rinaldi@uniroma1.it (V.R.); marialudovica.costanzo@uniroma1.it (M.L.C.); rocco.pasqua@uniroma1.it (R.P.); loiacono.2068496@studenti.uniroma1.it (F.L.); piergaspare.palumbo@uniroma1.it (P.P.); vito.dandrea@uniroma1.it (V.D.); giulio.illuminati@uniroma1.it (G.I.); 2 Department of Cardiac Surgery, Sapienza University of Rome, Viale Regina Elena, 324, 00161 Rome, Italy; fabio.miraldi@uniroma1.it; 3 Department of Interventional Cardiology, Sapienza University of Rome, Viale Regina Elena, 324, 00161 Rome, Italy; gaetano.tanzilli@uniroma1.it

**Keywords:** simultaneous PCI and EVAR, CAD, AAA, combined approach

## Abstract

**Background**: Performing percutaneous coronary intervention (PCI) and endovascular aneurysm repair (EVAR) at the same time represents a groundbreaking development in the multidisciplinary treatment of cardiovascular disease. This combined PCI–EVAR approach bridges a critical gap by offering treatment for patients who have both coronary artery disease and aortic aneurysms. This innovative strategy exemplifies the evolving landscape of cardiovascular care, providing a new solution for complex clinical situations that previously required separate procedures. **Methods**: Six patients with critical coronary artery lesions and asymptomatic infrarenal aortic aneurysms (AAAs) ≥ 6 cm diameter, as well as one patient with critical coronary artery lesions and endoleak type 1A with aneurysms ≥ 6 cm, underwent simultaneous coronary artery revascularization through percutaneous intervention (PCI) and endovascular aneurysm repair (EVAR). The occurrence of any intraoperative or postoperative complication was considered to be the primary endpoint of the study, including the abortion or failure of either PCI or EVAR, bleeding requiring a conversion to open surgical procedures, the failure of local anesthesia, postoperative myocardial or lower limb ischemia, and a postoperative serum creatinine level of >125 mmol/L or of >180 mmol/L in patients affected by chronic renal failure. The overall length of the procedure, X-ray exposure, the quantity of iodine contrast medium administered, and the length of recovery were considered to be secondary endpoints. **Results**: Postoperative complications included two episodes of acute renal failure in the two patients already affected by chronic renal failure, which were easily resolved with adequate daily hydration and the elimination of nephrotoxic drugs. In no cases did cardiac ischemia or lower limb ischemia occur. The average procedure duration was 198 min (range: 180–240 min), the average fluoroscopy duration was 41.7 min (range: 35–50 min), the average amount of iodinated contrast medium was 34.8 mL (range: 30–40 mL), and the mean length of hospitalization was 2.7 days (range: 2–5 days). **Conclusions**: In selected patients, this surgical approach has demonstrated safety, reduced hospitalization times, minimized risks associated with complications from the untreated condition if procedures were performed at different times, and facilitated the effective management of intraoperative complications due to the presence of a multidisciplinary team. However, the limited number of patients necessitates further research.

## 1. Introduction

The coexistence of common risk factors and underlying biological mechanisms creates a strong link between abdominal aortic aneurysm (AAA) and coronary artery disease (CAD). Studies have shown that 40% to 60% of patients with AAA also have CAD [1,2]. Additionally, heart attacks (acute myocardial infarction) contribute to nearly half of all deaths associated with AAA surgery (both during and after the operation) [3], and major heart attack is a common complication during the first 5 years after elective EVAR [4]. In patients with CAD, it is therefore always important to assess for the presence of AAA using ultrasound. Conversely, it is crucial to test for CAD, even if asymptomatic, in patients with AAA [5,6]. The coexistence of significant CAD and AAA requiring surgical repair necessitates a multidisciplinary evaluation to determine the appropriate treatment strategy. Traditionally, surgical interventions for these two conditions were performed at separate times, necessitating the prioritization of the condition to be treated and the timing between the two surgeries. This approach exposed patients to the risk of complications from the untreated condition [7,8]. The combined surgical approach for treating significant CAD and AAA has been documented in case reports and typically involves open surgery for at least one of the two conditions, with favorable postoperative outcomes [9,10,11]. The simultaneous performance of percutaneous coronary intervention (PCI) and endovascular aneurysm repair (EVAR) marks a pioneering advancement in the multidisciplinary treatment of cardiovascular diseases. The concurrent PCI and EVAR approach fills a significant gap in treating patients with co-existing coronary and aortic pathologies. This innovative strategy exemplifies the evolving nature of cardiovascular care, offering a novel solution to complex clinical scenarios that were previously addressed in multiple, separate interventions. Cardiovascular diseases, including both coronary and aortic pathologies, represent a significant global health burden, contributing to substantial morbidity and mortality worldwide. Traditionally, the management of these conditions has often necessitated separate interventions tailored to each specific anatomical site of disease. However, emerging evidence suggests that a unified approach to treating concurrent coronary and aortic diseases may offer advantages in terms of both efficacy and patient outcomes.

## 2. Materials and Methods 

From January 2021 to March 2023, six patients with critical coronary artery lesions and asymptomatic infrarenal aortic aneurysms (AAA) ≥ 6 cm diameter, as well as one patient with critical coronary artery lesions and endoleak type 1A with aneurysms ≥ 6 cm diameter, underwent simultaneous coronary artery revascularization through percutaneous intervention (PCI) and endovascular aneurysm repair (EVAR). The patient with type 1A endoleak underwent a first intervention 7 years before the onset of endoleak, which consisted of positioning an aortobiiliac endograft with a suprarenal free-flow stent (Bolton Medical, Sunrise, FL, USA). The patient had an abdominal aortic ultrasound every 6 months, and the most recent scan showed an increase in the size of the aneurysm sac (from 5.3 to 6.1 cm in 8 months). As a result, the patient underwent an abdominal aortic Computed Tomography (CT) angiography, which confirmed the presence of blood flow to the aneurysm sac from the proximal graft attachment. The baseline characteristics and risk factors of the patients’ series are listed in Table 1. Informed consent for the combined procedure was obtained from all the patients. The patients’ records were evaluated retrospectively; therefore, institutional ethics committee approval for the study was waived. The decision for the simultaneous treatment of both lesions was taken on a case-by-case basis, after a multidisciplinary staff meeting to discuss each patient and consider essentially the critical status of coronary arteries and aneurysmal diameter ≥ 6 cm. The multidisciplinary staff included the vascular surgeons in charge of the patients, the cardiac surgeons, the interventional cardiologists, and the anesthesiologists. Critical coronary lesions were considered as lesions >75% larger than at the previous coronary angio-CT scan, either with an unstable aspect during imaging, symptomatic, or associated with stable symptoms such as effort angina or dyspnea and with an ejection fraction of >50% during echocardiogram. In two patients with chronic kidney disease, preparation was carried out according to the company protocol, with the administration of 250 cc of Sol bicarbonate 1.4% at 100 mL/h 1 h before the contrast medium procedure and at 80 mL/h immediately after the procedure in one patient with GFR < 60 mL/min and >40 mL/min, and with hydration using 1000 cc of 0.9% physiological solution intravenously in one patient with GFR between 60 and 90 mL/min. One patient had an allergy to iodinated contrast medium and, 3 days before the procedure, underwent desensitizing therapy with Prednisone at a dose of 25 mg 1 cp × 2/day; this was reduced to 1 cp/day for two days after the procedure and 1 cp/day for the following two days and then suspended. Also, the patient was given Ranitidine at a dose of 150 mg 1 cp/day from three days before the procedure, which was suspended the day following the procedure. No allergic episodes occurred during the contrast medium procedure. All the procedures were performed in the interventional cardiology suite, which was also equipped for femoral artery cutdown procedures such as transcatheter aortic valve implantations (TAVIs), with an anesthesiological assistance throughout the whole procedure. The patients underwent the combined treatment while under oral single antiplatelet treatment (aspirin at 100 mg/day or clopidogrel at 75 mg/day) and oral statin treatment (athorvastatin at 20 mg/day) for at least a week. During PCI, a clopidogrel charge was performed. The post-treatment medical treatment included a dual antiplatelet and statin regimen, which continued for six months, associated with subcutaneous low-molecular-weight heparin (enoxaparin, at 2000 IU twice a day) for a week. All the procedures were performed percutaneously using the same femoral artery puncture, under local anesthesia. Significant coronary lesions involved the right coronary artery and circumflex in two patients, the right and anterior descending in another patient, the right coronary artery in two patients, the descending anterior in one patient, and the circumflex in the last one. During coronary angiography, there are innovative techniques, such as optical coherence tomography (OCT), intravascular ultrasound (IVUS), and quantitative coronary angiography (QCA), employed in specific cases where there is diagnostic ambiguity or uncertainty about the stenting outcome. Optical coherence tomography (OCT) allows for the evaluation of coronary plaque composition, stent strut apposition, and neointimal hyperplasia, providing high-resolution images with the ability to differentiate between lipid-rich and fibrous plaque. However, it has a limited depth of penetration and is susceptible to motion artifacts. Intravascular ultrasound (IVUS) is useful for assessing lumen size, plaque burden, and stent expansion. It provides cross-sectional images of the coronary artery, allowing for the accurate measurement of vessel dimensions. However, its resolution is lower than OCT, and it requires the use of a larger catheter. Quantitative coronary angiography (QCA) permits the measurement of lesion severity, the assessment of fractional flow reserve (FFR), and the evaluation of stent expansion. However, it only provides two-dimensional images and may underestimate lesion severity in complex lesions. The limitations of these techniques also include availability, additional cost, and time [12,13,14].

In the present case series, they were not utilized. Stenosis severity and stent deployment success were determined solely by angiography. In one case, a patient with significant stenosis of the right coronary and circumflex arteries had prompted the use of FFR to assess the significance of the circumflex stenosis, as it appeared questionable on angiography. This stenosis was found to be significant and was treated. All patients demonstrated a thrombolysis in myocardial infarction (TIMI) 3 flow postoperatively. The TIMI scale assigns a value from 0 to 3 to the blood flow observed during angiography, the results of this assessment are shown in Table 2.

Coronary angiography and PCI were performed first, then introducer sheaths were changed for the EVAR procedure, which was performed with ultrasound assistance, as previously described. EVAR always consisted of the insertion of an aortobiiliac endograft with a suprarenal free-flow stent (Bolton Medical, Sunrise, FL, USA) or of a prosthetic cuff with free flow positioned at the ostium of the renal arteries (Bolton Medical, Sunrise, FL, USA). The hemostasis of the femoral access was always obtained via a Perclose ProGlide device (Perclose ProGlide, Abbott Cardiovascular, Chicago, IL, USA), except in one patient with PAD; due to uncontrolled right femoral artery bleeding, there was the need of surgical preparation of the femoral artery and direct suturing with Prolene 5-0. IVUS (Volcano Visions PV 8.2F; Volcano Japan Inc., Tokyo, Japan) was used in all patients to identify landmarks for correct stent-graft placement (lowest renal artery ostium, aortic bifurcation, origin of hypogastric arteries). No patient had an aneurysm extending to the inter- or suprarenal aorta. In one case, the aneurysm extended to the left hypogastric artery, so a prosthetic branch with distal hook in the external iliac artery was placed and the left hypogastric artery itself was embolized. Postoperatively the patients were monitored using their electrocardiogram ( EKG), serum troponin level, and an assessment of the femoral puncture site after 24 h. They were discharged home on postoperative day two, in the event of an uneventful postoperative course. A control angio-CT scan of the thoraco-abdominal aorta was obtained at one month after the procedure, and subsequent vascular and cardiologic controls were performed according to current protocols.

### Study’s Endpoints

The occurrence of any intraoperative or postoperative complication was considered to be the primary endpoint of the study, including the abortion or failure of either PCI or EVAR, bleeding requiring a conversion to open surgical procedures, the failure of local anesthesia, postoperative myocardial or lower limbs ischemia, and a postoperative serum creatinine level of >125 mmol/L or of >180 mmol/L in patients affected by chronic renal failure. The overall length of the procedure, X-ray exposure, the quantity of iodine contrast medium administered, and the length of hospitalization were considered to be secondary endpoints.

## 3. Results

All the patients were males of a mean age of 71.5 years (range: 67 to 75 years). Three patients had symptoms suggesting an underlying coronary artery disease and, prior to hospitalization for the treatment of AAA, underwent a coronary artery angio-CT scan showing a significant stenosis, respectively, of the right, anterior descending, and circumflex coronary artery. The decision for a simultaneous PCI and EVAR was taken prior to hospitalization for a planned EVAR, in order not to delay EVAR itself, due to the consistent potential risk of a rupture of the AAA during the interval between the two procedures. During PCI, the above-mentioned coronary arteries were treated using angioplasty and drug-eluting stenting. The remaining four patients had stable symptoms, suggesting coronary artery stenosis, so, before the recovery for the aneurysm treatment, they underwent a coronary artery angio-CT scan showing significant stenosis. All these patients presented with single or double vessels disease, which were located, respectively, in the right and circumflex arteries in two patients, the right and anterior descending arteries in another patient, and in the right coronary artery in the last one. One intraoperative complication occurred in one patient; this was uncontrolled bleeding from the right femoral artery in a patient with PAD that was adequately resolved through surgical preparation of the femoral artery and direct suturing with Prolene 5-0. However, this procedure required the patient to be intubated under general anesthesia, and required the surgical procedure to be extended by 40 min. Postoperative complications included two episodes of acute renal failure in the two patients already affected by chronic renal failure, which were easily resolved with adequate daily hydration and the elimination of nephrotoxic drugs. In no cases did cardiac ischemia or lower limb ischemia occur (Table 3). The average procedure duration was 198 min (range: 180–240 min), the average fluoroscopy duration was 41.7 min (range: 35–50 min), the average amount of iodinated contrast medium was 34.8 mL (range: 30–40 mL), and the mean length of hospitalization was 2.7 days (range: 2–5 days) (Table 4).

## 4. Discussion

The dual procedural approach of performing both percutaneous coronary intervention (PCI) and endovascular aneurysm repair (EVAR) in a single session presents a complex but interesting strategy in the management of patients with concurrent cardiovascular conditions. The advantages and disadvantages of endovascular cardiac and aortic procedures are reported in Table 5.

When both conditions coexist, the one with complications should be treated first. However, when both pathologies are asymptomatic or paucisymptomatic, the literature reports cases wherein coronary artery disease was treated first, as acute myocardial infarction can occur in 60–70% of cases during the preoperative period [8,15,16]. In some cases, in a patient with multiple coronary vessels disease and AAA > 6 cm or aortic dissection, a simultaneous approach was used, combining coronary artery by-pass surgery (CABG) and EVAR [9,10,17].

One of the most compelling advantages of performing both percutaneous coronary intervention (PCI) and endovascular aneurysm repair (EVAR) in a single operative session is the notable efficiency gained; the patients benefit from a singular period of preparation, anesthesia, and recovery, rather than undergoing these phases multiple times. This approach not only minimizes the logistical burden on both the healthcare facility and the patient but also reduces the patient’s exposure to the risk of complications from the untreated condition, compared to staged surgeries performed at different times. The reduced total operative time can decrease the stress response to surgery, potentially leading to better overall patient outcomes. Furthermore, the shorter cumulative recovery period allows patients to return more quickly to their daily lives, which is particularly beneficial for elderly or frail patients who might have a harder time recovering from multiple surgical interventions. Moreover, the efficiency of this dual procedural approach also extends to hospital resource utilization. By handling two major interventions at once, the hospital can optimize the use of operating rooms and potentially reduce the waiting times for other patients needing surgical or interventional care.

The strategy of combining PCI and EVAR in a single session provides immediate readiness for complications, which is a crucial advantage in handling the complexities of concurrent cardiovascular conditions. This integrated approach allows the medical team to swiftly manage any arising issues during the intervention, a critical factor given the interdependencies of the conditions being treated. In traditional setups where interventions are performed separately, switching from one procedure to another may involve significant time lags and additional preparation. However, in a combined session, all necessary equipment and expertise are available at once, which significantly reduces the response time to any unforeseen events. The presence of a multidisciplinary team, including vascular surgeons, cardiologists, and anesthesiologists, in the operating room provides a robust platform for immediate and effective decision-making [18].

Enhancing the management of contrast medium in PCI and EVAR procedures not only safeguards against renal complications but also ensures the effectiveness of the treatment. Through the integration of IVUS and meticulous planning, the team was able to reduce contrast dependency significantly, relying on high-resolution images for accurate interventions [19,20]. Moreover, this approach allows for adaptive contrast administration, where adjustments can be made based on real-time responses, providing a tailored and safer procedure for patients with kidney vulnerabilities.

The challenge posed by the small sample size in our study is not uncommon in specialized medical research, especially when dealing with complex procedures like combined PCI and EVAR. The small number of participants inherently restricts the power of the study to detect a true effect and makes it difficult to generalize the results to a broader population.

The use of a more concentrated contrast medium in combined PCI and EVAR procedures does indeed elevate the risk of acute kidney injury (AKI), particularly in patients with pre-existing conditions such as chronic kidney disease (CKD). However, as observed in our study, this risk can be effectively managed and mitigated through specific strategies. Our findings indicated that, despite the increased risk associated with using a more concentrated contrast medium, only two instances of AKI occurred, and they involved patients who already had CKD. Importantly, these cases of AKI were managed successfully through rigorous hydration protocols and the temporary cessation of nephrotoxic drugs [21]. The risk of bleeding, a critical concern in any surgical procedure, can be heightened due to the extensive nature of combined interventions. Implementing rigorous hemodynamic monitoring throughout the procedure allows for the immediate detection and management of any bleeding issues. These findings highlight the necessity of close monitoring and proactive management to optimize patient safety in these complex cardiovascular interventions [22].

The risk of infection can be mitigated by adhering to stringent sterile techniques during the procedure. This includes the use of barrier precautions, surgical site antisepsis, and appropriate antibiotic prophylaxis. Developing an antibiotic stewardship program that tailors antibiotic use to the specific needs of the procedure and patient can prevent overuse and help manage the risk of antibiotic-resistant infections. Although combined procedures are inherently longer, efforts should be made to minimize the time the patient spends under anesthesia and in surgery. This can be achieved through meticulous preoperative planning, ensuring all necessary equipment and personnel are ready and available before the procedure begins. The assessment at the femoral puncture site after 24h was also critical to ensure there were no complications such as hematoma or pseudoaneurysm, common issues associated with femoral artery access. This rigorous monitoring helps in the early detection and management of complications that could potentially escalate if left unnoticed. These strategies can reduce the incidence of complications.

The insights derived from this study must be approached with prudence due to its retrospective design, small and heterogeneous sample size, and the absence of a control group. These limitations may reduce the strength and generalizability of its conclusions, even if the data were meticulously analyzed to ensure an objective interpretation. These methodological constraints underscore the need for more robust investigations.

It is imperative that future research be directed towards conducting larger-scale, prospective trials to corroborate these preliminary findings and help refine these combined procedural protocols further. Investigating the long-term efficacy and patient satisfaction post-procedure will also be crucial to fully ascertain the benefits and potential risks associated with this treatment approach. This future research will be vital in establishing robust guidelines that can be universally recommended for managing complex multi-system vascular diseases.

## 5. Conclusions

Simultaneous PCI and EVAR have demonstrated safety and efficacy in a patient demographic burdened with concurrent coronary and aortic diseases. When treating certain conditions, delaying surgery can lead to additional complications. This approach allows for addressing multiple issues during a single procedure, minimizing the risks associated with waiting for separate surgeries. Having a multidisciplinary team readily available in the operating room allows for a faster and more coordinated response if any unforeseen complications arise during surgery. This can significantly improve patient outcomes. The approach promises a swift return to daily activities, thereby improving the quality of life. However, limitations of the study are its retrospective nature and the limited number of participants. While the results are promising, more research with a larger patient population is needed to definitively confirm the safety and efficacy of this surgical approach.

## Figures and Tables

**Table 1:** Demographics and risk factor.

Variable	
Male gender, n (%)	7 (100)
Age (years)	71.5 (67–75)
Mean body mass index	24.3 (23–26)
Hypertension, n (%)	5 (71.4)
Smoking, n (%)	6 (85.7)
Symptomatic coronary artery disease, n (%)	3 (42.8)
Dyslipidemia, n (%)	5 (71.4)
Diabetes, n (%)	2 (28.6)
Peripheral arterial disease, n (%)	1 (14.3)
Chronic renal disease, n (%)	2 (28.6)

**Table 2:** Thrombolysis in myocardial infarction (TIMI) scale.

	Meaning
TIMI 0	No blood flow, the artery is completely occluded.
TIMI 1	Minimal collateral flow, insufficient to adequately perfuse the myocardium.
TIMI 2	Partial flow, with slow and incomplete filling of the vessel.
TIMI 3	Normal flow, the vessel is completely open and blood flows freely.

**Table 3:** Primary outcomes.

Results	n (%)
Unsuccessful PCI	-
Unsuccessful EVAR	-
Bleeding conversion	1 (14.3)
Failure of local anesthesia	-
Postoperative MI	-
Postoperative lower limbs ischemia	-
Postoperative creatinine blood levels > 125 mmol	-
Postoperative creatinine blood levels > 180 mmol	2 (28.6)

PCI, percutaneous coronary intervention; EVAR, endovascular aneurysm repair; MI, myocardial infarction.

**Table 4:** Secondary outcomes.

Results	Mean (Range)
Length of the procedure (minutes)	198 (180–240)
Length of fluoroscopy (minutes)	41.7 (35–50)
Quantity of contrast medium (mL)	34.8 (30–40)
Length of hospitalization (days)	2.7 (2–5)

**Table 5:** Advantages and disadvantages of endovascular cardiac and aortic procedures.

	Advantages	Disadvantages
PCI + stenting	Percutaneous access; Local anesthesia; Do not require extracorporeal circulation; Ability to treat multiple lesions; Cost-effective, with rapid recovery and shorter hospitalizations.	X-ray exposure; Administration of contrast medium; Restricted indications for patients with a common trunk or triple-vessel disease; Dual antiplatelet therapy.
EVAR	Percutaneous access; Local anesthesia; Reduced operative times; Reduced blood loss; Less severe and shorter-lasting postoperative pain; Shorter hospital stays; Reduced risk of infection; Ability to treat high-risk patients.	Not all patients with an abdominal aortic aneurysm are candidates for EVAR; X-ray exposure; Administration of contrast medium; Risk of endoleak; May require additional procedures; Expensive procedure (the economic impact is, however, minimal, given the absence of the need for intensive care, fewer severe complications, shorter hospital stays, and a faster recovery); Risk of the graft moving out of position.
Simultaneous PCI + stenting and EVAR	Resolution of both coronary and abdominal aortic aneurysm issues in a single surgical session; Local anesthesia; Use of the same arterial access for both procedures; Shorter hospital stays; Cost-effective, with rapid recovery and shorter hospitalizations; Both specialists will be present in the operating room in case either condition complicates, allowing for a rapid intervention.	Longer operative times compared to the two separate procedures; Higher contrast dose and radiation exposure during a single procedure; A complication from PCI and stenting may necessitate rescheduling the EVAR; Selected patients.

## Data Availability

The analyzed data used in this study are available upon reasonable request.
